# Early-life famine exposure and subsequent risk of chronic diseases in later adulthood: a population-based retrospective cohort study

**DOI:** 10.3389/fpubh.2024.1495296

**Published:** 2025-01-07

**Authors:** Rui Zhao, Qi Zheng, Le-qin Chen

**Affiliations:** School of Physical Education, Shanxi Normal University, Taiyuan, China

**Keywords:** chronic diseases, later adulthood, China’s great famine, famine, multimorbidity

## Abstract

**Background:**

Over the past few decades, China has experienced significant demographic and epidemiological changes. The sharp decline in fertility and mortality rates has accelerated population aging, contributing to an increase in the prevalence of chronic diseases. The nutritional condition during early life is associated with the onset of chronic illnesses later in adulthood. However, it remains unclear whether this association also increases the risk of multimorbidity in later adulthood.

**Objectives:**

This study aimed to systematically evaluate the association between early-life famine exposure and the subsequent development of 14 chronic diseases, as well as multimorbidity, and whether there exists a discrepancy in gender, residence, and famine severity.

**Methods:**

Data are from the 2018 Wave 4 CHARLS database, a national survey covering 19,816 participants aged 45 years or older. Drawing from our past research on famine in China, we incorporated 3,867 participants, categorizing them into three groups based on their birth years. Considering that climate conditions and population density can influence the intensity of famine, we characterize its severity by establishing a threshold of 50% excess death rate. The 14 chronic diseases assessed by CHARLS were used as the main outcome indicator, with multimorbidity as a secondary outcome indicator. We conducted a stepwise logistic regression analysis to investigate the impact of exposure to famine in early life affects the development of chronic diseases in adulthood, presenting the findings as ORs and 95% CIs. Additionally, we assess multiple moderating factors such as gender, residence, and famine severity to evaluate the outcomes.

**Results:**

Of the 3,867 participants included, the prevalence of each chronic disease ranged from 1.1% (Psychiatric disease) to 10.7% (Hypertension). Overall, 27.0% of participants reported being affected by at least one chronic disease, while 9.6% indicated they had suffered from two or more chronic conditions. Early-life exposure to famine makes it more likely to develop diabetes in later adulthood OR (95% CI) of 1.85 (1.26 to2.72), stroke OR (95% CI) of 1.96 (1.17 to 3.29), kidney disease OR (95% CI) of 1.91 (1.07 to 3.40), and multimorbidity OR (95% CI) of 1.39 (1.08 to 1.80), compared to those who did not face such conditions. The moderating effects analysis revealed that less severe famine exposure during toddlerhood was associated with an increased risk of multimorbidity in adulthood OR (95% CI) of 1.43 (1.01 to 2.03). Males exposed to famine during toddlerhood had a higher risk of multimorbidity in adulthood OR (95% CI) of 2.26 (1.29 to 3.98). Compared to the unexposed group, rural residents who experienced famine exposure in their early life are at a higher risk of developing multimorbidity by about 1.5 times in adulthood.

**Conclusion:**

Experiencing famine in early childhood increases susceptibility to developing chronic diseases in later adulthood, with the risk of diabetes, stroke, and kidney disease nearly doubling. The association of multimorbidity in later adulthood varies according to the severity of the famine, gender, and residential area.

## Introduction

China’s Great Famine was one of the most severe humanitarian crises of the mid-20th century, occurring between 1959 and 1961, with the estimated death toll during this period ranged from 15 million to 45 million people (1). This catastrophic event not only caused immediate loss of life but also contributed to long-term changes in the country’s demographic and health patterns. Over the past few decades, China has experienced significant demographic and epidemiological changes. The sharp decline in fertility and mortality rates has accelerated population aging, contributing to an increase in the prevalence of chronic diseases (2, 3). From 1990 to 2010, early childhood mortality decreased by nearly 80%, and the proportion of infectious diseases in the overall disease burden fell to just 10%. Concurrently, non-communicable diseases, particularly cardiovascular and musculoskeletal disorders, have emerged as the leading contributors to disease burden (4). These shifts accelerated the transition from infectious diseases to chronic diseases in terms of public health. Chronic diseases affect not only individuals and their families but also create a substantial financial strain on society (5). Recent studies have increasingly pointed out that the nutritional condition in early life is associated with the onset of chronic illnesses in later adulthood (6, 7). However, it remains unclear whether this association also increases the risk of multimorbidity in later adulthood.

The Developmental Origins of Health and Disease (DOHaD) hypothesis suggests that adverse experiences during childhood are crucial determinants of long-term health outcomes (8). Evidence from the famine in Ukraine (1932–1933) in 16 Soviet republics (128,225 cases of diabetes) suggests that famine exposure during pregnancy is associated with an increased risk of developing diabetes in adulthood (9). Similarly, a study on the cohort affected by the 1959–1962 Chinese famine found a link between early-life famine exposure and diabetes (10). Additionally, similar associations have been observed for other chronic diseases, including kidney disease, asthma, and arthritis (7, 11, 12). Genetic studies have corroborated these findings that research on individuals born during China’s famine era analyzed DNA methylation in the IGF2 gene and lipid levels, revealing that early-life famine exposure significantly affects adult lipid levels, even after adjusting for confounding factors such as age and gender (13). However, current research has largely overlooked the potential interconnections among the pathways of multimorbidity, as well as the possibility that different chronic diseases may coexist.

Multiple studies indicate that multimorbidity can reduce quality of life, cause disability, shorten lifespan, and increase mortality rates (14, 15). In Germany, researchers using data from two cross-sectional KORA-Age studies found that individuals exposed to famine during early life were more likely to develop multimorbidity in adulthood (16). In China, research has shown that adverse childhood experiences (like physical abuse or domestic violence), can leading a higher risk for multimorbidity (17). However, the evidence connecting famines to multimorbidity is scarce outside of these studies. Additionally, there is no evidence demonstrating a direct association between the Chinese Famine and multimorbidity outcomes.

In this research, we employed the China Health and Retirement Longitudinal Study (CHARLS) database, which is a longitudinal cohort that represents the national population and focuses on individuals aged 45 and older. Our study examined information on 14 chronic disease indicators among Chinese adults. The primary aim was to investigate how early childhood exposure to famine, particularly during infancy and toddlerhood, We also considered moderating factors such as gender, residence, and famine severity to assess their impact on the results. Additionally, we aim to provide empirical evidence for the prevention and management of chronic illnesses in older adults, thereby facilitating the process of healthy aging.

## Methods

### Study design and participants

**Figure 1:**
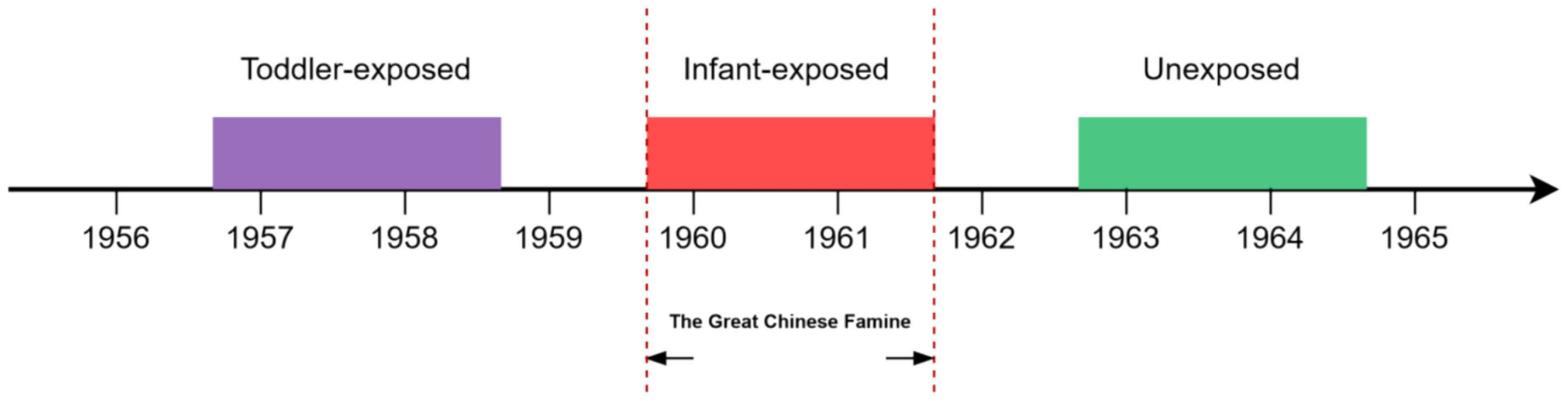
The classification of famine exposure groups based on birth dates.

Data are from the 2018 Wave 4 CHARLS database, a national survey covering aged 45 years or older in 28 of mainland China’s 31 provinces (excluding Tibet, Ningxia, and Hainan) (18). A multistage probability sampling method was employed to select participants, and data on chronic diseases were collected through face-to-face interviews using computer-assisted personal interviewing techniques (Supplementary Methods 1; Supplementary Table 2). More detailed description of the sampling design and data quality of the CLHRLS has been reported elsewhere (19). Additionally, we excluded participants who lacked birth year data, had incomplete personal information, or had no record of chronic diseases. Drawing from our past research on famine in China, we incorporated a total of 3,867 participants, categorized into three groups based on their birth years (Figure 1). The group exposed during infancy consisted of 904 individuals born from Oct 1, 1959, to Sep 30, 1961. The toddler exposure included 1,255 individuals born from Oct 1, 1956, to Sep 30, 1958. The Unexposed consisted of 1,708 individuals born from Oct 1, 1962, to Sep 30, 1964. This study has received approval from the Ethics Review Committee of Peking University (No. IRB00001052-11015).

### Assessment of famine exposure and severity

The Great Famine from 1959 to 1962 affected all of mainland China, but its impact differed among provinces due to variations in climate, population density, and regional food policies. As a result, we define the intensity of famine by measuring the excess death rate (EDR) in each province (20). First, we determined the province of each subject using the community Identity (ID) variable from the Primary Sampling Units (PSU) (21). We match the population status of each province in the China Statistical Yearbook to calculate the EDR (22). Second, we established a cutoff point of 50% EDR (23). Provinces with an EDR falling below this level were categorized as having less severe famine exposure, whereas those with an EDR at or above this threshold were categorized as severely affected famine areas (Supplementary Methods 2).

### Outcomes

Chronic diseases and multimorbidity were used as outcome variables. Multimorbidity is typically defined as the presence of at least two of the 14 chronic conditions in a single individual (24). The 2018 CHARLS survey collected data on these 14 chronic diseases through self-reports of physician diagnoses. Each participant was asked about their status (yes or no) regarding conditions such as hypertension, dyslipidemia, diabetes, cancer, chronic lung disease, liver disease, heart disease, stroke, kidney disease, digestive disorders, mental illness, memory-related diseases, arthritis, and asthma (Supplementary Table 2).

### Assessment of covariates

Covariates reflecting the participants’ status were collected via questionnaires, including gender, education level, marital status, residential area, Physical Activity (PA), smoking status, and drinking habits (Supplementary Table 1).

We used participants’ reports on the types and duration of PA and used the midpoint of each time interval to calculate the average daily duration (25). Based on the guidelines of the International Physical Activity Questionnaire (IPAQ), we calculated the weekly level of physical activity (26). Finally, we divided the weekly level of physical activity into three categories: low intensity, moderate intensity, and high intensity (27) (Supplementary Methods 3).

### Statistical analysis

Categorical variables were represented using counts and percentages (for example, gender, region, and education), while continuous variables were described by their means and standard deviations (such as age). We measure the severity of famine using a 50% EDR (less severely <50% EDR, severely ≥50% EDR).

First, we checked for multicollinearity among variables using the Variance Inflation Factor (VIF) and selected covariates for model refitting using LASSO regression. Subsequently, we employed a stepwise logistic regression to investigate the impact of famine exposure on the emergence of chronic diseases in later adulthood. We constructed stepwise logistic regression models: Model 1 crude model; Model 2 accounted for variables such as gender, geographic location, education level, marital status, and severity of famine (Remove the subgroups analysis variables themselves); and Model 3 included additional adjustments for PA levels, smoking habits, and alcohol consumption.

We conducted stratified analyses based on famine severity (less severe, severe), gender (male, female), and geographic region (rural, urban) to examine the potential differences in outcomes for chronic diseases and multimorbidity across different moderating factors. Considering the impact of social activities and per capita income on chronic diseases, we performed sensitivity analyses in which we added the social activity and per capita income variables to the original multivariate correction model. Data analysis was conducted using R version 4.4.0 (R Foundation for Statistical Computing, Vienna, Austria). The findings are reported as Odds Ratios (OR) with corresponding 95% Confidence Intervals (95% CIs), and a *p*-value less than 0.05 was considered statistically significant.

## Results

Among the 3,867 participants included in the study, 1,976 (51.1%) were females, and 1,891 (48.9%) were males. The prevalence of each chronic disease ranged from 1.1% (Psychiatric disease) to 10.7% (Hypertension; Supplementary Table 1). In total, 27.0% of the participants reported having been affected by at least one chronic disease, while 9.6% had encountered two or more chronic conditions (Supplementary Figure 1).

Our research suggests that people who experienced famine during their early years, particularly in infancy and toddler stages, generally exhibited lower educational achievements, decreased PA levels, and an increased propensity for smoking compared to those who did not endure such conditions (Table 1).

**Table 1:** The characteristics of the study population.

Characteristic	Overall	Unexposed	Infant-exposed	Toddler-exposed
Sample size, N (%)	3,867	1,708(44.1)	904(23.3)	1,255(32.4)
Age (years)				
Gender, N (%)				
Male	1,891(48.9)	827(48.4)	422(46.7)	642(51.2)
Female	1,976(51.1)	881(51.6)	482(53.3)	613(48.8)
Age	57.5 ± 2.7	54.8 ± 0.7	57.8 ± 0.7	60.8 ± 0.7
Regions, N (%)				
Rural	2,819(72.9)	1,238(72.5)	654(72.3)	927(73.9)
Urban	1,019(26.4)	455(26.6)	244(27.0)	320(25.5)
Other	29(0.7)	15(0.9)	6(0.7)	8(0.6)
Education level, N (%)				
Primary school or lower	1,915(49.5)	796(46.6)	415(45.9)	704(56.1)
Junior high school	1,169(30.2)	611(35.8)	242(26.8)	316(25.2)
Senior high school	627(16.2)	222(13.0)	208(23.0)	197(15.7)
University and above	156(4.0)	79(4.6)	397(4.3)	38(3.0)
Marital Status, N(%)				
Married/Cohabiting	3,564(84.4)	1,453(85.1)	751(83.1)	1,060(84.5)
Widowed/Single/Divorced/Separated	603(15.6)	255(14.9)	153(16.9)	195(15.5)
Per capita income, CNY	6,375.8 ± 12998.7	6,175.5 ± 12742.1	5,607.3 ± 10633.0	7,169.3 ± 14,676.3
PA, N (%)				
Low	1,708(44.2)	707(41.4)	426(47.1)	575(45.8)
Middle	1,896(49.0)	882(51.6)	414(45.8)	600(47.8)
High	263(6.8)	119(7.0)	64(7.1)	80(6.4)
Social activity, N (%)	2,410(62.3)	1,086(63.6)	567(62.7)	757(60.3)
Smoking, never, No. (%)	2,202(56.9)	1,003(58.2)	526(58.2)	673(53.6)
Drinking habits, N (%)				
Never drinking	2,441(63.2)	1,064(62.1)	571(63.2)	810(64.6)
≤1 time/month	303(7.8)	143(8.4)	66(7.3)	94(7.5)
>1 time/month	1,120(29.0)	504(29.5)	267(29.5)	349(27.9)
Self-reported health, N (%)				
good	979(25.3)	443(25.9)	234(25.9)	302(24.1)
fair	1,862(48.2)	842(49.3)	424(46.9)	596(47.5)
poor	833(21.5)	335(19.6)	196(21.7)	302(24.1)
Famine severity, N (%)				
Less severely	2,223(57.5)	922(54.0)	569(62.9)	732(58.3)
Severely	1,644(42.5)	786(46.0)	335(37.1)	523(41.7)

Continuous data are reported as the mean (SD), and categorical data are reported as the number and percentage of participants. N, sample size. CNY, Chinese Yuan.

Table 2 demonstrates the connection between varying stages of famine exposure and the probability of developing multimorbidity in adulthood. Research indicates that individuals who faced famine during their early years are at a higher risk for chronic health issues, such as hypertension, diabetes, cancer, strokes, kidney diseases, and memory-related disorders, in contrast to those who did not encounter famine in their childhood. After accounting for confounding factors, there remains a correlation: diabetes OR (95% CI) of 1.85 (1.26 to 2.72), stroke OR (95% CI) of 1.96 (1.17 to 3.29), and kidney disease OR (95% CI) of 1.91 (1.07–3.40), relative to non-exposed individuals. Sensitivity analyses revealed that these results remained largely consistent (Supplementary Tables 3, 4).

**Table 2:** Association between famine and subsequent chronic diseases in adulthood.

	Model 1: Crude model OR (95% CI)			Model 2: Adjusted model OR (95% CI)		
	Group 0	Group 1	Group 2	Group 0	Group 1	Group 2
Hypertension	Ref	1.08(0.83–1.42)	**1.29(1.02–1.64)**	Ref	1.09(0.77–1.54)	1.17(0.86–1.61)
Dyslipidemia	Ref	0.86(0.65–1.13)	1.03(0.81–1.30)	Ref	0.87(0.61–1.23)	1.12(0.82–1.52)
Diabetes	Ref	1.00(0.70–1.44)	**1.43(1.06–1.94)**	Ref	1.43(0.92–2.24)	**1.85(1.26–2.72)**
Cancer	Ref	**2.18(1.06–4.49)**	1.46(0.71–3.04)	Ref	2.15(0.89–5.17)	1.57(0.66–3.76)
Chronic lung disease	Ref	1.03(0.70–1.52)	1.14(0.81–1.60)	Ref	1.28(0.79–2.06)	0.91(0.57–1.48)
Liver disease	Ref	0.75(0.48–1.19)	0.70(0.46–1.06)	Ref	0.77(0.44–1.34)	0.80(0.49–1.32)
Heart disease	Ref	0.97(0.68–1.38)	1.21(0.89–1.63)	Ref	0.96(0.62–1.50)	1.28(0.88–1.88)
Stroke	Ref	1.43(0.93–2.19)	**1.80(1.24–2.62)**	Ref	1.63(0.92–2.90)	**1.96(1.17–3.29)**
Kidney disease	Ref	**1.79(1.15–2.79)**	1.20(0.77–1.89)	Ref	**1.91(1.07–3.40)**	1.23(0.68–2.23)
Digestive disease	Ref	1.15(0.84–1.59)	1.11(0.83–1.49)	Ref	1.30(0.88–1.94)	1.28(0.89–1.85)
Psychiatric disease	Ref	0.94(0.42–2.11)	1.21(0.62–2.39)	Ref	0.74(0.30–1.83)	1.09(0.52–2.26)
Memory-related disease	Ref	**2.87(1.38–5.99)**	2.06(0.99–4.28)	Ref	2.45(0.99–6.03)	1.95(0.81–4.71)
Arthritis	Ref	1.14(0.85–1.53)	0.87(0.65–1.15)	Ref	1.28(0.89–1.83)	0.88(0.61–1.27)
Asthma	Ref	1.19(0.65–2.20)	1.11(0.63–1.96)	Ref	1.62(0.74–3.57)	1.03(0.46–2.29)

Group 0, Unexposed; Group 1, Infant-exposed; Group 2, Toddler-exposed; OR, odds ratio; 95% CI, 95% confidence intervals; Ref, reference. Model 1: crude model. Model 2 was adjusted for Gender, Residence, Education, Marital status, Severity, PA, Smoking status, and Drinking habits. The bold values indicate statistically significant.

Table 3 evaluates the association between experiencing famine in early childhood and the increased likelihood of developing multimorbidity in later adulthood. After Adjusting for confounding variables, the effects remain significant (Supplementary Table 5).

**Table 3:** Association between famine and multimorbidity in the overall study population and subgroups.

	Model 1: Crude model OR (95% CI)			Model 2: Adjusted model OR (95% CI)		
	Group 0	Group 1	Group 2	Group 0	Group 1	Group 2
Overall study population^*^	Ref	1.07(0.85–1.34)	**1.30(1.07–1.59)**	Ref	1.31(0.99–1.74)	**1.39(1.08–1.80)**
Severity^#^						
Less severely	Ref	1.19(0.88–1.61)	**1.35(1.02–1.78)**	Ref	1.37(0.94–2.00)	**1.43(1.01–2.03)**
Severely	Ref	0.96(0.67–1.37)	1.28(0.96–1.71)	Ref	1.23(0.81–1.88)	1.34(0.92–1.95)
Gender^##^						
Male	Ref	0.89(0.62-1.27)	**1.39(1.05–1.85)**	Ref	1.68(0.86–3.29)	**2.26(1.29–3.98)**
Female	Ref	1.21(0.90–1.64)	1.23(0.93–1.62)	Ref	1.24(0.91–1.69)	1.23(0.0.92–1.65)
Regions^###^						
Rural	Ref	1.29(1.02-1.64)	**1.19(0.91–1.56)**	Ref	**1.47(1.05–2.05)**	**1.41(1.04–1.93)**
Urban	Ref	0.89(0.58–1.38)	1.36(0.94–1.96)	Ref	1.06(0.63–1.79)	1.38(0.87–2.20)

Group 0, Unexposed; Group 1, Infant-exposed; Group 2, Toddler-exposed; OR, odds ratio; 95% CI, 95% confidence intervals; Ref, reference. Model 1: crude model. The bold values indicate statistically significant.

* Model 2 was adjusted for Gender, Residence, Education, Marital status, Severity, PA, Smoking status, and Drinking_habits.

# Model 2 was adjusted for Gender, Residence, Education, Marital status, PA, Smoking status, and Drinking_habits.

## Model 2 was adjusted for Residence, Education, Marital status, Severity, PA, Smoking status, and Drinking_habits.

### Model 2 was adjusted for Gender, Education, Marital status, Severity, PA, Smoking status, and Drinking_habits.

Table 3 illustrates the general relationship between famine and multimorbidity, and the difference in this association across different moderating factors. Upon comparing severely famine-stricken areas, we have observed a correlation between toddler exposure to less severe famine and increased susceptibility to multimorbidity in adulthood,with an OR (95% CI) of 1.43 (1.20 to 2.03). When analyzing the data by gender, our research indicates that men who faced famine during their early childhood are 2.3 times more prone to experiencing multimorbidity in adulthood than those who did not encounter such circumstances, with an OR (95% CI) of 2.26 (1.29 to 3.98). Furthermore, the research has also revealed a significant urban–rural disparity in the association between adult multimorbidity and toddler exposure to famine. Individuals living in rural regions who faced famine during their early life exhibited a 1.5 times higher likelihood of developing multiple health conditions in adulthood compared to those not exposed. Additionally, we found that the findings related to individual chronic diseases were largely aligned with those concerning multimorbidity (Supplementary Tables 6–8).

## Discussion

This is the first study to explore the association between early-life famine and chronic diseases, as well as multimorbidity, in a nationally representative sample in China. We observed that adults who experienced famine early in life exhibit a heightened susceptibility to chronic diseases, with the risk of diabetes, stroke, and kidney disease increasing by nearly twofold. The risk of multimorbidity associated with early famine exposure varies according to the severity of the famine, gender, and residential area. Notably, rural residents and males exhibited significantly greater susceptibility. Additionally, exposure to even less severe famine during toddler was associated with increased multimorbidity in later adulthood.

Nutritional deficiencies during critical developmental periods, such as infancy and childhood, increase the risk of multiple diseases in adulthood (28, 29), which aligns with our primary findings. Our analysis reveals that experiencing famine in toddlers was associated with a 1.85-fold increased risk of developing diabetes in toddlerhood compared to the unexposed group. This finding corroborates earlier studies on famine exposure during childhood and adolescence and its link to adult-onset diabetes (30, 31). The main reason for this result is that famine causes physiological damage to multiple organs, including the pancreas and liver (32). Research has shown that diabetes, blood lipid levels, and hypertension are risk factors for kidney disease. Based on existing evidence, diabetes, hypertension, and blood lipids are risk factors for kidney disease (7). We study found a connection between early famine exposure and the development of kidney disease in adulthood. Infancy is a critical growth period, and inadequate nutrition during this stage can lead to diminished renal function or glomerular filtration rates (7, 33). However, our study only found an association between early famine exposure and hypertension in adulthood in crude models, but this association disappeared after adjusting for confounding variables. Previous studies on hypertension associated with early famine also reported similar evidence (34, 35). This could be affected by a range of different factors, such as birth weight, participant origin, and grouping criteria (36, 37). Similarly, animal studies have shown that malnutrition during fetal development is linked to dyslipidemia (38, 39). However, our study’s findings regarding dyslipidemia differ from previous research, possibly because participants received lipid-lowering treatments, leading to an underestimation of results. Furthermore, the “confounding effect” theory suggests that smoking and psychological stress can also affect blood lipids, which may partially explain our results (40).

Our results also showed that experiencing famine during toddlerhood is associated with a 1.96-fold increased risk of stroke in toddlerhood compared to unexposed groups. This finding aligns with research based on the China Kadoorie Biobank (CKB) regarding ischemic strokes (41), although it differs from two studies conducted in the Netherlands, possibly due to variations in environment, sample size, and famine group classifications (42, 43). In terms of asthma and chronic lung disease, our research findings suggest that individuals who experienced famine exposure during infancy may have a higher risk of asthma, but no such conclusion was drawn for chronic lung disease. This is consistent with another study based on the 2011 CHARLS database (11). The reason for this is that the in-utero growth restriction and low birth weight caused by famine exposure during infancy are associated with an increased risk of asthma in adulthood, and vitamin deficiency during infancy may also impact the development of the lungs and their function (44, 45).

Our study suggests that there is no significant link between experiencing famine during childhood and the risk of developing cancer in adulthood. This conclusion aligns with earlier studies, including those examining the effects of early famine on gastric cancer in Shanghai, China, as well as investigations into the link between early famine and cancer rates in the Netherlands (46, 47). However, investigations into specific cancer types remain limited. Our study did not observe a link between early life experiences of famine and rheumatic disease, which differs from previous findings (12). The misclassification of arthritis and the quality of an individual’s growing environment may contribute to bias in the results (48). Famine exposure during infancy might also be linked to digestive and memory-related diseases in later life. Although these relationships were not evident in our study, previous evidence suggests that even individuals born after October 1962, who did not experience the famine (our control group), may develop chronic diseases due to their parents’ famine experiences (49). Therefore, further research is required to investigate the link between early-life famine exposure and chronic diseases in adulthood, which requires global efforts to cover more representative population samples and establish lifelong health monitoring to provide reliable epidemiological evidence of this relationship.

Multimorbidity among the older adults currently poses a significant global health challenge. In China, the prevalence of multimorbidity in adults reaches up to 50% and may continue to grow at a rate of 1%, imposing a substantial burden on families and healthcare systems (24, 51). Our findings suggest that adult survivors who experienced famine as children are more prone to multimorbidity, indicating that modifying factors associated with multimorbidity could effectively reduce its incidence. Analysis of different moderating factors revealed that children exposed to less severe famine had a higher risk of developing multimorbidity in adulthood compared to those exposed to severe famine. This may be related to various factors, including the complex causes of death during the famine, which may include direct starvation or diseases and infections caused by malnutrition and deteriorating sanitary conditions. These causes of death are difficult to distinguish in statistics, affecting the accuracy of excess death rates. Our study categorized severity based on a 50% excess death rate, with regions like Anhui, Guizhou, and Qinghai experiencing rates far exceeding 100%, thus classified as severely affected. The increased mortality explains the heightened multimorbidity risk for toddlers exposed to mild famine.

Our study found that male adults are more likely to develop multimorbidity due to early-life famine exposure. This may be attributed to differing health risk factors at gender, such as the higher smoking and drinking rates among men in China, which are closely linked to chronic diseases (52). Furthermore, there is a significant urban–rural disparity in the impact of early famine exposure on multimorbidity. In rural areas, children who experience famine have an increased risk of multimorbidity. During the Great Famine, issues in the collectivization movement, natural disasters, and human factors led to reduced food production, with rural-to-urban migration restrictions exacerbating rural famine severity. The lagging health effects of famine contribute to higher multimorbidity risk in rural areas (53). Additionally, the faster aging rate and economic and healthcare disadvantages in rural areas compared to urban areas may further increase this risk. Therefore, it is crucial to implement proactive interventions and strengthen health monitoring and disease prevention efforts for chronic diseases. Given the increase in unhealthy lifestyles and aging, there is an urgent need to improve lifestyle behaviors and slow down aging to prevent chronic diseases (30).

This research has multiple advantages. To begin with, it is the first to investigate how early-life famine relates to chronic illnesses and multimorbidity, utilizing a nationally representative sample from China. Secondly, using large population-representative data and appropriate statistical analyses, we established a connection between exposure to famine in early life and the development of chronic diseases later on. In addition, our study provides a detailed analysis of the differences between multiple moderating factors and chronic diseases.

However, this study had several limitations. Firstly, a retrospective cohort study has some limitations in inferring causality. It can only identify an association but cannot directly prove causality. Secondly, the sampling design included participants from 28 of the 31 provinces in mainland China, and variations in famine severity across provinces may affect the results. Nevertheless, we conducted sensitivity analyses using different excess death rates. Thirdly, data collection through questionnaires combined with face-to-face computer-assisted personal interviews may introduce recall bias for some measures, such as PA. However, the large-scale national survey minimized errors as much as possible. Finally, not including migration data in our sample may lead to bias, but previous research suggests that provincial migration was restricted by policy, preventing individuals from moving across regions significantly affected by the famine (54, 55).

Our study shows that experiencing famine in early childhood increases susceptibility to developing chronic diseases in later adulthood, with the risk of diabetes, stroke, and kidney disease nearly doubling. The association of multimorbidity in later adulthood varies according to the severity of the famine, gender, and residential area. These findings underscore the long-term health consequences of early nutritional deprivation and highlight the importance of early intervention for individuals exposed to famine. Furthermore, this study advocates for incorporating early-life nutritional history into public health policies to reduce the future burden of multimorbidity and improve long-term health outcomes.

## Data Availability

The datasets presented in this study can be found in online repositories. The names of the repository/repositories and accession number(s) can be found in the article/Supplementary material.
