# Supplementary material for: Early-life famine exposure and subsequent risk of chronic diseases in later adulthood: a population-based retrospective cohort study
Source: Front Public Health. 2025 Jan 7;12:1495296. doi: 10.3389/fpubh.2024.1495296 (PMC11746111; doi:10.3389/fpubh.2024.1495296)
Supplement: Supplementary file 1 [file Data_Sheet_1.PDF]

# Supplementary appendix

Early-life famine exposure and subsequent risk of chronic diseases in later adulthood: A population-based retrospective cohort study

# Supplementary appendix

**eMethods 1** Study design and participants ........ 1

**eMethods 2** Excess death rate and area categories ........ 2

**eMethods 3** MET values and physical activity intensity categories ........ 3

**eTable 1** Harmonized definitions for key covariates in the study ........ 4

**eTable 2** Questionnaire items and prevalence of chronic diseases in CHARLS ........ 5

**eTable 3** Association between famine and subsequent chronic diseases in adulthood: additionally adjusted for social activity ........ 6

**eTable 4** Association between famine and subsequent chronic diseases in adulthood: adjusted for per capita income ........ 7

**eTable 5** Association between famine and multimorbidity in the overall study ........ 8

**eTable 6** Comparison of prevalence of chronic diseases between less severely and severely famine ........ 9

**eTable 7** Comparison of prevalence of chronic diseases between male and female ... 10

**eTable 8** Comparison of prevalence of chronic diseases between rural and urban ..... 11

**eFigure 1** The quantity and prevalence of chronic disease types ........ 12

**eEthics approval** ........ 13

**Funding** ........ 13

**eMethods 1 Study design and participants**

The CHARLS's baseline survey includes one person per household aged 45 years of age or older and their spouse, totaling 17,708 individuals, living in 10,257 households in 450 villages / urban communities (1). A stratified (by per capita GDP of urban districts and rural counties) multi-stage (county / district-village / communityhousehold) probability proportional to size (PPS) random sampling strategy was adopted.

At the first stage, all county-level units were sorted (stratified) by region, within the region by urban district or rural county, and by GDP per capita (Tibet was the only province not included). The region was a categorical variable based on the NBS division of provinces. After this sorting (stratification), 150 counties or urban districts were chosen with probability proportional to population size (2). For each county-level unit, 3 Primary Sampling Units (PSU) (villages and urban neighborhoods) are randomly chosen with probability proportional to population (3). Hence CHARLS is nationally representative and representative of both rural and urban areas within China. Counties and districts in 28 provinces are included in the CHARLS sample.

In light of the outdated household listings at the village/community level due to population migration, CHARLS designed a mapping/listing software (Charls-GIS) that makes use of Google-earth map images to list all dwelling units in all residential buildings to create sampling frames

(1) Zhao, Yaohui, Yisong Hu, James P Smith, John Strauss, and Gonghuan Yang. 2014a. "Cohort Profile: The China Health and Retirement Longitudinal Study (CHARLS)." Int J Epidemiol 43 (1):61–8.

(2) Zhao, Yaohui, Crimmins E. M., Peifeng (Perry) Hu, Yisong Hu, Tao Ge, Jung Ki Kim, John Strauss, Gonghuan Yang, Xiangjun Yin, and Yafeng Wang. 2014b. "China Health and Retirement Longitudinal Study 2011–2012 National Baseline Blood Data User's Guide." Tech. rep., National School of Development, Peking University.

(3) Zhao, Yaohui, John Strauss, Gonghuan Yang, John Giles, Peifeng (Perry) Hu, Yisong Hu, Xiaoyan Lei, Man Liu, Albert Park, James P. Smith, and Yafeng Wang. 2013. "China Health and Retirement Longitudinal Study: 2011-2012 National Baseline User's Guide." Tech. rep., National School of Development, Peking University

**eMethods 2 Excess death rate and area categories**

The Excess Death Rate refers to the difference between the normal death rate and the death rate during the famine period. This difference reflects the additional mortality burden caused by the famine. The Normal Death Rate is typically defined as the average death rate in the years leading up to the famine, serving as a baseline for comparison. The Death Rate During Famine refers to the mortality rate during the famine years.

For data preparation, death rate data is obtained from the "China Statistical Yearbook" for the years before and during the famine. Specifically, the period of includes the three years before the famine (1956-1958) and the three years during the famine (1959-1961). These data points are essential for calculating the normal and famine-related death rates, which are central to the subsequent analysis.

To calculate the Excess Death Rate, first, the Normal Death Rate is determined by computing the average death rate from 1956 to 1958. Then, the Death Rate During Famine is calculated as the average death rate for the years 1959 to 1961. The Excess Death Rate is then derived using the formula:

Excess Death Rate = (Death Rate During Famine−Normal Death Rate) / Normal Death Rate × 100%

Lastly, a threshold of 50% is used to classify the severity of the famine’s impact across different regions. Provinces with an excess death rate below 50% are classified as mildly affected by famine, while those with an excess death rate above 50% are classified as severely affected.

**eMethods 3 MET values and physical activity intensity categories**

According to the CHARLS questionnaire, the duration of daily physical activity types is divided into 5 categories (0 min, 10~29 min, 30~119 min, 120~239 min, ≥ 240 min), and the median is used to calculate the exercise time for high, moderate, and low-intensity physical activities. The weekly duration of physical activity = number of days performing each type of physical activity × duration of each type per day (1).

The amount of high-intensity physical activity is calculated using the Metabolic Equivalent (MET). MET is a measure of the intensity of physical activity. According to the International Physical Activity Questionnaire (IPAQ) assessment criteria, walking has a MET value of 3.3, moderate-intensity activity has a MET value of 4.0, and high-intensity activity has a MET value of 8.0 (2).

The score of physical activity is calculated by the formula: 8.0 × total duration of high-intensity activity per week + 4.0 × total duration of moderate-intensity activity per week + 3.3 × total duration of walking per week [15]. The weekly level of physical activity is categorized into low-intensity physical activity (< 600 METs/week), moderate-intensity physical activity (600~3,000 METs/week), and high-intensity physical activity (> 3,000 METs/week) (3).

(1) Zeng Z, Bian Y, Cui Y, Yang D, Wang Y, Yu C. Physical activity dimensions and its association with risk of diabetes in middle and older aged Chinese people. *Int J Environ Res Public Health*. 2020;17(21):7803. doi:10.3390/ijerph17217803

(2) Bai A, Tao L, Huang J, Tao J, Liu J. Effects of physical activity on cognitive function among patients with diabetes in China: A nationally longitudinal study. *BMC Public Health*. 2021;21(1):481. doi:10.1186/s12889-021-10537-x

(3) FAN MY, LÜ J. Chinese guidelines for data processing and analysis concerning the International Physical Activity Questionnaire. *Chin J Epidemiol*. 2014;35((8)):961-964.

**eTable 1 Harmonized definitions for key covariates in the study**

| Variables | Values |
|---|---|
| Education | |
| Primary school or lower | No formal education (illiterate)/Did not finish primary schoo/Sishu/home school/Elementary school/ |
| Junior high school | Middle school |
| Senior high school | High school/Vocational school |
| University and above | Two-/Three-Year College/Associate degree/Four-Year College/Bachelor's degree/Maste's degree/Doctoral degree/Ph.D. |
| Marital | |
| Married or partnered | Married and living together with |
| Single | Widowed/Single/Divorced/Separated |
| Cigarette smoking | |
| No | Never |
| Yes | Previous, and current |
| Alcohol drinking | |
| No | Never |
| Yes | ≤1 time/month<br>>1 time/month |
| Famine severity | |
| Less severely | The excess death rate is less than 50%. |
| Severely | The excess death rate is greater than 50%. |

**eTable 2 Questionnaire items and prevalence of chronic diseases in CHARLS**

| Types of Chronic Diseases | Questionnaire Items（codes：DA007） | Prevalence (%) |
|---|---|---|
| Hypertension | Have you been diagnosed with Hypertension by a doctor? | 10.7 |
| Dyslipidemia | Have you been diagnosed with Dyslipidemia (elevation of low density lipoprotein, triglycerides (TGs),and total cholesterol, or a low high density lipoprotein level) by a doctor? | 10.0 |
| Diabetes | Have you been diagnosed with Diabetes or high blood sugar by a doctor? | 5.8 |
| Cancer | Have you been diagnosed with Cancer or malignant tumor (excluding minor skin cancers) by a doctor? | 1.1 |
| Chronic lung disease | Have you been diagnosed with Chronic lung diseases, such as chronic bronchitis , emphysema ( excluding tumors, or cancer) by a doctor? | 4.7 |
| Liver disease | Have you been diagnosed with Liver disease (except fatty liver, tumors, and cancer) by a doctor? | 3.3 |
| Heart disease | Have you been diagnosed with Heart attack, coronary heart disease, angina, congestive heart failure, or other heart problems by a doctor? | 5.9 |
| Stroke | Have you been diagnosed with Stroke by a doctor? | 4.0 |
| Kidney disease | Have you been diagnosed with Kidney disease (except for tumor or cancer) by a doctor? | 3.0 |
| Digestive disease | Have you been diagnosed with Stomach or other digestive diseases (except for tumor or cancer) by a doctor? | 6.6 |
| Psychiatric disease | Have you been diagnosed with Emotional, nervous, or psychiatric problems by a doctor? | 1.1 |
| Memory-related disease | Have you been diagnosed with Emotional, nervous, or psychiatric problems by a doctor? | 1.2 |
| Arthritis | Have you been diagnosed with Arthritis or rheumatism by a doctor? | 7.4 |
| Asthma | Have you been diagnosed with Asthma by a doctor? | 1.7 |

**eTable 3 Association between famine and subsequent chronic diseases in adulthood: additionally adjusted for social activity**

| | Model 1 : Crude model OR (95% CI) | | | Model 2 : Adjusted model OR (95% CI) | | |
|---|---|---|---|---|---|---|
| | Group 0 | Group 1 | Group 2 | Group 0 | Group 1 | Group 2 |
| Hypertension | Ref | 1.08(0.83-1.42) | **1.29(1.02-1.64)** | Ref | 1.08(0.76-1.53) | 1.17(0.85-1.61) |
| Dyslipidemia | Ref | 0.86(0.65-1.13) | 1.03(0.81-1.30) | Ref | 0.87(0.62-1.24) | 1.12(0.83-1.52) |
| Diabetes | Ref | 1.00(0.70-1.44) | **1.43(1.06-1.94)** | Ref | 1.46(0.94-2.28) | **1.88(1.28-2.77)** |
| Cancer | Ref | **2.18(1.06-4.49)** | 1.46(0.71-3.04) | Ref | 2.18(0.91-5.24) | 1.57(0.66-3.76) |
| Chronic lung disease | Ref | 1.03(0.70-1.52) | 1.14(0.81-1.60) | Ref | 1.27(0.78-2.04) | 0.91(0.57-1.67) |
| Liver disease | Ref | 0.75(0.48-1.19) | 0.70(0.46-1.06) | Ref | 0.78(0.45-1.36) | 0.81(0.49-1.33) |
| Heart disease | Ref | 0.97(0.68-1.38) | 1.21(0.89-1.63) | Ref | 0.97(0.62-1.51) | 1.29(0.88-1.89) |
| Stroke | Ref | 1.43(0.93-2.19) | **1.80(1.24-2.62)** | Ref | 1.64(0.92-2.92) | **1.97(1.17-3.29)** |
| Kidney disease | Ref | **1.79(1.15-2.79)** | 1.20(0.77-1.89) | Ref | **1.87(1.05-3.34)** | 1.22(0.67-2.21) |
| Digestive disease | Ref | 1.15(0.84-1.59) | 1.11(0.83-1.49) | Ref | 1.32(0.89-1.95) | 1.28(0.89-1.86) |
| Psychiatric disease | Ref | 0.94(0.42-2.11) | 1.21(0.62-2.39) | Ref | 0.74(0.30-1.85) | 1.09(0.52-2.27) |
| Memory-related disease | Ref | **2.87(1.38-5.99)** | 2.06(0.99-4.28) | Ref | 2.38(0.96-5.88) | 1.94(0.80-4.70) |
| Arthritis | Ref | 1.14(0.85-1.53) | 0.87(0.65-1.15) | Ref | 1.28(0.89-1.84) | 0.88(0.61-1.27) |
| Asthma | Ref | 1.19(0.65-2.20) | 1.11(0.63-1.96) | Ref | 1.67(0.76-3.68) | 1.04(0.47-2.32) |

Note: Group0, Unexposed; Group1, Infant-exposed; Group2, Toddler-exposed; OR, odds ratio; 95% CI, 95% confidence intervals; Ref, reference. Model 1: crude model. Model 2 was adjusted for Gender, Residence, Education, Marital status, Severity, PA, Smoking status, and Drinking habits, Social activity. The bold values indicate statistically significant.

**eTable 4 Association between famine and subsequent chronic diseases in adulthood: adjusted for per capita income**

| | Model 1 : Crude model OR (95% CI) | | | Model 2 : Adjusted model OR (95% CI) | | |
|---|---|---|---|---|---|---|
| | Group 0 | Group 1 | Group 2 | Group 0 | Group 1 | Group 2 |
| Hypertension | Ref | 1.09(0.83-1.46) | 1.34(1.03-1.72) | Ref | 1.21(0.76-1.92) | 1.40(0.92-2.12) |
| Dyslipidemia | Ref | 0.84(0.61-1.15) | 1.05(0.80-1.36) | Ref | 1.07(0.67-1.69) | 1.36(0.91-2.04) |
| Diabetes | Ref | 1.00(0.66-1.51) | **1.55(1.11-2.17)** | Ref | 1.28(0.71-2.29) | 1.52(0.92-2.53) |
| Cancer | Ref | **1.99(0.94-4.19)** | 1.37(0.65-2.89) | Ref | 2.03(0.69-5.95) | 1.14(0.37-3.50) |
| Chronic lung disease | Ref | 1.18(0.79-1.75) | 1.12(0.78-1.60) | Ref | 1.55(0.83-2.89) | 0.85(0.44-1.64) |
| Liver disease | Ref | 0.82(0.51-1.34) | 0.76(0.49-1.18) | Ref | 0.77(0.37-1.59) | 0.91(0.49-1.70) |
| Heart disease | Ref | 1.06(0.73-1.54) | 1.18(0.85-1.64) | Ref | 0.87(0.49-1.57) | 1.27(0.78-2.07) |
| Stroke | Ref | 1.31(0.80-2.14) | **1.85(1.22-2.79)** | Ref | 1.63(0.71-3.73) | **2.49(1.22-5.09)** |
| Kidney disease | Ref | **1.79(1.10-2.89)** | 1.27(0.79-2.03) | Ref | 1.65(0.48-5.66) | 1.48(0.46-4.79) |
| Digestive disease | Ref | 1.18(0.82-1.69) | 1.21(0.88-1.67) | Ref | 1.28(0.76-2.17) | 1.38(0.85-2.44) |
| Psychiatric disease | Ref | 1.27(0.55-2.94) | 1.08(0.49-2.38) | Ref | 0.21(0.03-1.67) | 1.74(0.71-4.26) |
| Memory-related disease | Ref | **3.74(1.58-8.87)** | **3.12(1.35-7.20)** | Ref | 2.01(0.39-10.41) | **4.58(1.21-17.42)** |
| Arthritis | Ref | 1.08(0.78-1.49) | 0.92(0.68-1.25) | Ref | 1.28(0.80-2.06) | 0.94(0.59-1.51) |
| Asthma | Ref | 1.35(0.66-2.75) | 1.30(0.68-2.49) | Ref | 2.53(0.65-9.87) | 2.19(0.60-7.97) |

Note: Group0, Unexposed; Group1, Infant-exposed; Group2, Toddler-exposed; OR, odds ratio; 95% CI, 95% confidence intervals; Ref, reference. Model 1: crude model. Model 2 was adjusted for Gender, Residence, Education, Marital status, Severity, PA, Smoking status, and Drinking habits, per capita income. The bold values indicate statistically significant.

## eTable 5 Association between famine and multimorbidity in the overall study

| | Multimorbidity OR (95% CI) | | |
|---|---|---|---|
| | Unexposed | Infant-exposed | Toddler-exposed |
| Model 1 | Ref | 1.07(0.85-1.34) | **1.30(1.07-1.59)** |
| Model 2 | Ref | 1.11(0.88-1.40) | **1.31(1.07-1.61)** |
| Model 3 | Ref | 1.31(0.99-1.73) | **1.39(1.08-1.80)** |

Note: OR, odds ratio; 95% CI, 95% confidence intervals; Ref, reference. OR represents the probability ra tio of developing multimorbidity chronic diseases for individuals who were exposed to famine, compared with those who were not exposed to famine, at 95% CIs. Model 1: crude model. Model 2: adjusted for Gender,Residence, Education,Marital status,Severity Model 3: further adjusted for PA,Smoking status, Drink ing habits based on Model 2 The bold values indicate statistically significant.

.

**eTable 6 Comparison of prevalence of chronic diseases between less severely and severely famine**

| | Less severely OR (95% CI) | | | Severely OR (95% CI) | | |
|---|---|---|---|---|---|---|
| | Group 0 | Group 1 | Group 2 | Group 0 | Group 1 | Group 2 |
| Hypertension | Ref | 1.02(0.64-1.61) | 1.13(0.74-1.73) | Ref | 1.20(0.71-2.05) | 1.22(0.76-1.96) |
| Dyslipidemia | Ref | 0.86(0.54-1.37) | 1.27(0.85-1.92) | Ref | 0.90(0.53-1.53) | 0.99(0.63-1.58) |
| Diabetes | Ref | 1.14(0.59-2.21) | **2.05(1.19-3.52)** | Ref | 1.72(0.93-3.17) | 1.67(0.96-2.91) |
| Cancer | Ref | 1.54(0.37-6.73) | 0.90(0.19-4.18) | Ref | 2.82(0.90-8.79) | 2.12(0.72-6.28) |
| Chronic lung disease | Ref | 1.17(0.63-2.16) | 0.91(0.50-1.67) | Ref | 1.55(0.71-3.37) | 0.90(0.41-1.96) |
| Liver disease | Ref | 0.66(0.31-1.39) | 0.60(0.30-1.22) | Ref | 0.94(0.41-2.14) | 1.10(0.54-2.24) |
| Heart disease | Ref | 1.13(0.63-2.05) | 1.38(0.80-2.37) | Ref | 0.87(0.44-1.71) | 1.20(0.70-2.08) |
| Stroke | Ref | 1.71(0.75-3.89) | **2.69(0.28-1.56)** | Ref | 1.65(0.73-3.74) | 1.38(0.63-3.03) |
| Kidney disease | Ref | 2.23(1.01-4.94) | 1.85(0.84-4.06) | Ref | 1.57(0.66-3.76) | 0.68(0.25-1.83) |
| Digestive disease | Ref | 1.42(0.86-2.34) | 1.27(0.78-2.07) | Ref | 1.16(0.61-2.21) | 1.37(0.78-2.39) |
| Psychiatric disease | Ref | 1.30(0.39-4.37) | 2.16(0.77-6.04) | Ref | 3.78(0.08-1.82) | 4.09(0.11-1.53) |
| Memory-related disease | Ref | 1.80(0.56-5.79) | 1.58(0.51-4.84) | Ref | 3.71(0.86-15.90) | 2.77(0.65-11.86) |
| Arthritis | Ref | 1.44(0.90-2.30) | 0.68(0.40-1.16) | Ref | 1.03(0.58-1.83) | 1.12(0.67-1.86) |
| Asthma | Ref | 1.34(0.46-3.94) | 1.49(0.56-3.95) | Ref | 1.95(0.60-6.36) | 0.41(0.08-2.06) |

Note: Group0, Unexposed; Group1, Infant-exposed; Group2, Toddler-exposed; OR, odds ratio; 95% CI, 95% confidence intervals; Ref, reference.

Model was adjusted for Gender, Residence, Education, Marital status, PA, Smoking status, and Drinking habits..

The bold values indicate statistically significant.

## eTable 7 Comparison of prevalence of chronic diseases between male and female

| | Male OR (95% CI) | | | Female OR (95% CI) | | |
|---|---|---|---|---|---|---|
| | Group 0 | Group 1 | Group 2 | Group 0 | Group 1 | Group 2 |
| Hypertension | Ref | 1.80(0.89-3.66) | **1.90(1.05-3.45)** | Ref | 0.94(0.63-1.41) | 1.00(0.68-1.45) |
| Dyslipidemia | Ref | 0.72(0.30-1.75) | 1.93(0.99-3.74) | Ref | 0.90(0.61-1.13) | 1.01(0.71-1.43) |
| Diabetes | Ref | 0.95(0.38-2.34) | **2.20(1.09-4.45)** | Ref | 1.46(0.88-2.41) | **1.69(1.08-2.66)** |
| Cancer | Ref | 5.82(0.46-73.34) | 1.43(0.08-24.10) | Ref | 1.79(0.69-4.61) | 1.55(0.62-3.89) |
| Chronic lung disease | Ref | 1.57(0.58-4.26) | 1.28(0.53-3.13) | Ref | 1.19(0.69-2.05) | 0.78(0.44-1.37) |
| Liver disease | Ref | 0.65(0.13-3.37) | 1.56(0.54-4.52) | Ref | 0.78(0.43-1.40) | 0.67(0.37-1.19) |
| Heart disease | Ref | 0.76(0.23-2.50) | 1.25(0.51-3.01) | Ref | 1.04(0.65-1.68) | 1.31(0.86-2.01) |
| Stroke | Ref | **3.38(1.05-10.92)** | 1.48(0.45-4.90) | Ref | 1.35(0.69-2.67) | **2.12(1.19-3.77)** |
| Kidney disease | Ref | 2.08(0.65-6.72) | 0.92(0.27-3.06) | Ref | 1.86(0.95-3.64) | 1.29(0.65-2.58) |
| Digestive disease | Ref | 1.78(0.77-4.14) | 1.20(0.53-2.68) | Ref | 1.20(0.77-1.88) | 1.31(0.87-1.98) |
| Psychiatric disease | Ref | 0.94(0.42-2.11) | 1.21(0.62-2.39) | Ref | 0.75(0.32-2.00) | 0.98(0.45,2.14) |
| Memory-related disease | Ref | 1.67(7.86-360.0) | 6.01(0.38-94.70) | Ref | 2.05(0.77-5.43) | 1.74(0.67-4.50) |
| Arthritis | Ref | 1.15(0.50-2.66) | 0.62(0.27-1.43) | Ref | 1.31(0.88-1.97) | 0.98(0.65-1.48) |
| Asthma | Ref | 1.19(0.10-14.20) | 2.01(0.33-14.76) | Ref | 1.66(0.72-3.83) | 0.82(0.33-2.03) |

Note: Group0, Unexposed; Group1, Infant-exposed; Group2, Toddler-exposed; OR, odds ratio; 95% CI, 95% confidence intervals; Ref, reference.

Model was adjusted for Residence, Education, Marital status, Severity, PA, Smoking_status, and Drinking_habits..

The bold values indicate statistically significant.

## eTable 8 Comparison of prevalence of chronic diseases between rural and urban

| | Rural OR (95% CI) | | | Urban OR (95% CI) | | |
|---|---|---|---|---|---|---|
| | Group 0 | Group 1 | Group 2 | Group 0 | Group 1 | Group 2 |
| Hypertension | Ref | 1.23(0.82-1.85) | 1.25(0.86-1.82) | Ref | 0.85(0.43-1.69) | 1.01(0.55-1.82) |
| Dyslipidemia | Ref | 0.96(0.63-1.46) | 1.18(0.82-1.72) | Ref | 0.65(0.34-1.25) | 1.02(0.59-1.77) |
| Diabetes | Ref | **2.05(1.21-3.46)** | **2.38(1.49-3.80)** | Ref | 0.57(0.22-1.46) | 1.02(0.49-2.09) |
| Cancer | Ref | 1.89(0.69-5.19) | 1.06(0.36-3.11) | Ref | 3.00(0.46-19.30) | 4.07(0.76-21.77) |
| Chronic lung disease | Ref | 1.65(0.96-2.86) | 1.01(0.57-1.78) | Ref | 0.46(0.15-1.43) | 0.71(0.28-1.79) |
| Liver disease | Ref | 0.94(0.48-1.87) | 1.00(0.54-1.83) | Ref | 0.61(0.23-1.60) | 0.50(0.20-1.27) |
| Heart disease | Ref | 0.81(0.45-1.46) | 1.37(0.85-2.19) | Ref | 1.28(0.64-2.56) | 1.19(0.62-2.31) |
| Stroke | Ref | 1.61(0.80-3.24) | 1.43(0.74-2.76) | Ref | 1.68(0.61-4.64) | **2.97(1.23-7.17)** |
| Kidney disease | Ref | 1.70(0.79-3.66) | 1.21(0.56-2.59) | Ref | 2.59(1.04-6.47) | **1.27(0.47-3.38)** |
| Digestive disease | Ref | 1.48(0.93-2.36) | 1.51(0.98-1.49) | Ref | 0.96(0.45-2.03) | 0.81(0.38-1.73) |
| Psychiatric disease | Ref | 1.14(0.36-3.58) | 1.16(0.41-3.24) | Ref | 0.42(0.09-2.07) | 1.17(0.40,3.44) |
| Memory-related disease | Ref | 2.43(0.91-6.49) | 1.78(0.67-4.75) | Ref | 2.90(0.25-33.35) | 3.28(0.33-32.64) |
| Arthritis | Ref | 1.19(0.78-1.81) | 0.73(0.47-1.13) | Ref | 1.58(0.77-3.26) | 1.51(0.775-3.00) |
| Asthma | Ref | 1.92(0.80-4.63) | 0.87(0.87-2.29) | Ref | 1.09(0.17-7.23) | 1.52(0.31-7.45) |

Note: Group0, Unexposed; Group1, Infant-exposed; Model was adjusted for Gender, Education, Marital status, Severity, PA, Social activity, Smoking status, and Drinking habits..

The bold values indicate statistically significant.

**eFigure 1 The quantity and prevalence of chronic disease types**

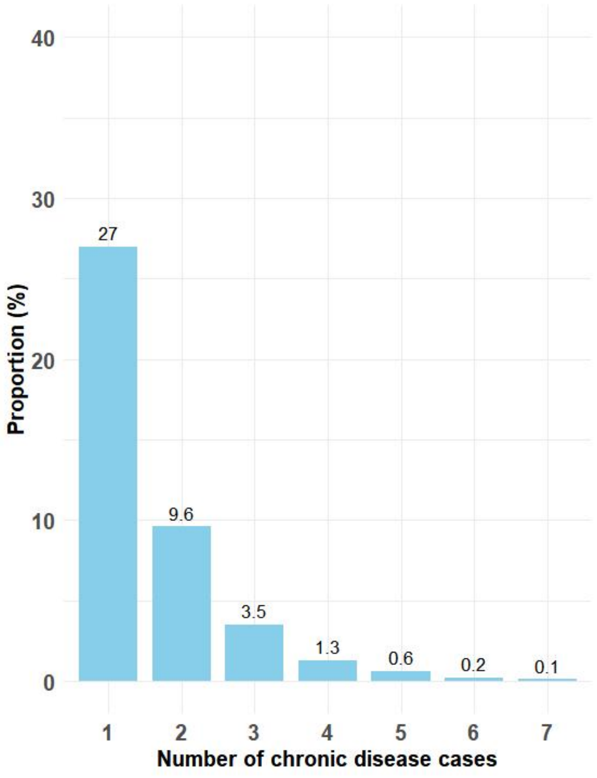

**eEthics approval**

Ethical approval for all the CHARLS waves was granted from the Institutional Review Board at Peking University. The IRB approval number for the main household survey, including anthropometrics, is IRB00001052-11015; the IRB approval number for biomarker collection, was IRB00001052-11014.

During the fieldwork, each respondent who agreed to participate in the survey was asked to sign two copies of the informed consent, and one copy was kept in the CHARLS office, which was also scanned and saved in PDF format. Four separate consents were obtained: one for the main fieldwork, one for the non-blood biomarkers and one for the taking of the blood samples, and another for storage of blood for future analyses.

**Funding**

The National Natural Science Foundation of China (grant number 70773002, 70910107022, 71130002, and 71450001), Behavioral and Social Research division of the National Institute on Aging of the National Institutes of Health in the United States (grant numbers 1R21AG031372, 1R01AG037031, R03TW008358RO3AG049144, R01AG053228), the World Bank Group (contract number 7145915, 71592347172961), Chinese medical board (contract number 13-154, 16-249), and Peking University all provided critical financial support for our project.
